# Editorial for the Special Issue: “The Issue of Multidrug-Resistant Pathogens in Nosocomial Infections”

**DOI:** 10.3390/antibiotics12121683

**Published:** 2023-11-30

**Authors:** Alberto Enrico Maraolo

**Affiliations:** First Division of Infectious Diseases, Cotugno Hospital, AORN Ospedali dei Colli, 80131 Naples, Italy; albertomaraolo@mail.com

Antimicrobial resistance (AMR) is a global problem; in 2019, before the Coronavirus Disease 2019 (COVID-19) pandemic, it was responsible of more deaths than any other infectious diseases, including human immunodeficiency virus and malaria [1]. The pandemic itself promoted AMR through several mechanisms. Although the guidelines recommended the empirical use of antibiotics only when there was a strong suspicion of a bacterial co-infection or superinfection, and not as a standard therapeutic measure for viral diseases [2], excessive antimicrobial agent use by COVID-19 patients was described, despite the relatively low rates of co-infections or secondary infections from bacteria [3]. Moreover, the pandemic diverted resources and disrupted antimicrobial stewardship programs.

Since AMR is supposed to represent a constant pandemic in the XXI century, it is mandatory to accrue knowledge about the epidemiology, risk factors, prognosis, an therapeutic management, especially in nosocomial settings, where AMR is more frequent.

This Special Issue includes different types of articles: comprehensive and systematic reviews with or without meta-analyses, totaling five full research articles written by researchers from all over the world. Overall, they reflect a vast array of experiences regarding nosocomial infections; moreover, the authors take stock of the situation concerning difficult-to-treat pathogens or infection prevention and control.

An overview of the published papers is offered below.

The only meta-analysis was published by Maraolo and colleagues, focusing on infections by *Stenotrophomonas maltophilia* [4]. Although its epidemiological impact is inferior to that of other Gram-negative pathogens, the threaten posed by *S. maltophilia* is growing, shored up by the unique combination of several resistance mechanisms [5] and by its propensity to affect fragile individuals, e.g., immunosuppressed subjects requiring intensive care or long-stay patients [6]. The treatment of *S. maltophilia* is not informed by high-quality evidence due to the difficulties in running randomized controlled trials (RCTs) including a sufficient number of cases, but it is mainly based on trimethoprim/sulfamethoxazole (TMP/SMX), although with much controversy about the role of potential alternatives and a combination regimen [7]. Maraolo et al. comprehensively assessing the available literature, included 24 observational studies, and found that TMP/SMX was associated with a higher mortality rate compared with that of fluoroquinolones; the results were statistically significant, although with a high prediction interval hinting at a high heterogeneity level [4]. This finding was influenced by the large study run by Sarzinsky and colleagues in the United States, likely including many cases of non-severe infections, since the mortality rate was lower than expected from the previous literature [8]. Some other interesting findings include that there were no statistically significant differences between TMP/SMX and the tetracycline derivatives as well as between the monotherapies and combination regimens [4]. Concerning for RCTs (e.g., NCT05575427, comparing minocycline as monotherapy against a combination with either levofloxacin or TMP/SMX), the jury is still out in defining the best treatment for *S. maltophilia* infections, but this meta-analysis questions the dogma of TMP/SMX as a therapeutic cornerstone [4].

The second systematic review revolves around another non-fermenting Gram-negative bacterium, *Pseudomonas aeruginosa* [9]. The researchers compared the rates of *P. aeruginosa* bloodstream infections (BSI) prior to and during the COVID-19 pandemic [10]. The key finding was an incremental increase in the incidence of *P. aeruginosa* bacteremia during the COVID-19 pandemic, likely explained by the high burden of long-length-of-stay patients or those admitted to intensive care units (ICU), as showed in several works, wherein *P. aeruginosa* stand out as the most relevant pathogen among the COVID-19 patients in an ICU [11]. The authors from Singapore actually had performed their research moving from the hypothesis that infection prevention and control (IPC) measures implemented concomitantly to the SARS-CoV-2 pandemic could on the contrary decrease the incidence of *P. aeruginosa* BSI.

In this respect the review from Schinas and coworkers is timely and exhaustive, providing practical recommendations on how to adopt effective measures aimed at decreasing and preventing the transmission of resistant pathogens in ICUs [12].

Gram-negative pathogens were the subject also of some research articles in this Special Issue.

For instance, Dalfino and collaborators gave an interesting account of a single-center, retrospective experience of ventilator-associated pneumonia (VAP) caused by carbapenem-resistant *Acinetobacter baumannii* (CRAB) in a non-COVID-19 ICU in southern Italy [13]. The Italian researchers included 90 cases and, by using a sound methodology to mitigate the biases linked with the observational nature of the study, showed the positive impact of cefiderocol-based therapy on mortality over that of the colistin-based regimens [13]. These findings corroborate the wisdom from observational studies on the effectiveness of cefiderol against CRAB [14], which contrasts the mixed results from RCTs [15]. Beyond the never-ending controversy about monotherapy versus a combination regimen, it is very likely due to the fact that the pharmacokinetic/pharmacodynamic features are to be taken into account to deliver the optimal treatment in critically ill patients with VAP [16].

Shifting to fermentative Gram-negative bacteria, the ROCAS study group (the acronym stands for “The Argentine Group for the Study of Bacteremia in Cancer and Stem Cell Transplant”) developed a score to define the risk of BSI posed by carbapenem-resistant *Enterobacterales* (CRE) in cancer and hematopoietic stem cell transplantation patients [17]. The ensuing score was quite simple, based only the length of antibiotic exposure, the length of hospitalization, and colonization by KPC. Interestingly, the negative predictive value if none of these elements were present was quite high, and it was equal to 98.1% [17]. This score, beyond its simplicity, which fits with the principles of Occam’s razor, derives its strengths from having a larger sample size than those of the previous studies on other well-known predictive models [18,19].

A study from Japan compared the outcomes of persistent BSI caused by Gram-negative bacteria, which were either multidrug-resistant or not, and fermenters or non-fermenters [20], in the further analysis of a previous published cohort [21]. Performing follow-up blood cultures (FUBCs) is matter of debate when BSI is related to Gram-negative pathogens, whereas it is considered an element of good clinical practice when it comes to *Staphylococcus aureus* [22]. The literature on FUBC is often characterized by numerous biases, hindering proper interpretation; the immortal time bias is the paramount example [23]. At any rate, according to common wisdom and some evidence, FUBC may be justified in Gram-negative BSI in fragile populations and in case of resistant pathogens as a causative agent [14]. Kitaya and coworkers found that AMR impacted negatively the prognosis in the case of persistent BSI caused by *Enterobacterales*; on the contrary, no differences were detected when non-fermenting Gram-negative pathogens were involved [20].

The same group of researchers investigated staphylococcal BSI as well, caused by both *S. aureus* and coagulase-negative staphylococci (CoNS), focusing on a subset of persistent infection and assessing the role of methicillin resistance [24]. The results were as follows: the methicillin-susceptible strains were associated with a lower mortality rate as far as the *S. aureus* infections were concerned, whereas in the CoNS group, no impact on prognosis stemmed from the mechanism of resistance [24]. Persistent BSI remains a great challenge for every physician; in the case of *S. aureus,* the first and foremost issue is the correct definition of persistence, from which derives the timing of FUBC, with an earlier time frame being more appropriate to repeat testing (24–48 h) than a period up to seven days (the one in the study) [25].

Last but not the least, a French group led by Lemmonier evaluated the trend in either Gram-negative or Gram-positive resistant pathogens over the 2018–2022 period; although, in general, no particular changes were observed, there were some increases and decreases in the prevalence of the given pathogens [26]. Worryingly, a rise in metallo-beta-lactamases detection was registered among *Enterobacterales* [26].

In conclusion, this Special Issue collects comprehensive reviews and research articles from scientists and clinicians affiliated with institutions located in three continents, reflecting the worldwide impact of AMR, especially in hospital settings.

## Data Availability

No research data were generated for this editorial that commented on already published articles.

## References

[1] Antimicrobial Resistance Collaborators (2022). Global burden of bacterial antimicrobial resistance in 2019: A systematic analysis. Lancet.

[2] Bassetti M., Giacobbe D.R., Bruzzi P., Barisione E., Centanni S., Castaldo N., Corcione S., De Rosa F.G., Di Marco F., Gori A. (2021). Clinical management of adult patients with COVID-19 outside intensive care units: Guidelines from the Italian Society of Anti-Infective Therapy (SITA) and the Italian Society of Pulmonology (SIP). Infect. Dis. Ther..

[3] Lai C.C., Chen S.Y., Ko W.C., Hsueh P.R. (2021). Increased antimicrobial resistance during the COVID-19 pandemic. Int. J. Antimicrob. Agents.

[4] Maraolo A.E., Licciardi F., Gentile I., Saracino A., Belati A., Bavaro D.F. (2023). *Stenotrophomonas maltophilia* Infections: A Systematic Review and Meta-Analysis of Comparative Efficacy of Available Treatments, with Critical Assessment of Novel Therapeutic Options. Antibiotics.

[5] Chang Y.T., Lin C.Y., Chen Y.H., Hsueh P.R. (2015). Update on infections caused by *Stenotrophomonas maltophilia* with particular attention to resistance mechanisms and therapeutic options. Front. Microbiol..

[6] Mojica M.F., Humphries R., Lipuma J.J., Mathers A.J., Rao G.G., Shelburne S.A., Fouts D.E., Van Duin D., Bonomo R.A. (2022). Clinical challenges treating *Stenotrophomonas maltophilia* infections: An update. JAC-Antimicrob. Resist..

[7] Kullar R., Wenzler E., Alexander J., Goldstein E.J.C. (2022). Overcoming *Stenotrophomonas maltophilia* Resistance for a More Rational Therapeutic Approach. Open Forum Infect. Dis..

[8] Sarzynski S.H., Warner S., Sun J., Matsouaka R., Dekker J.P., Babiker A., Li W., Lai Y.L., Danner R.L., Fowler V.G. (2022). Trimethoprim-Sulfamethoxazole versus Levofloxacin for *Stenotrophomonas maltophilia* Infections: A Retrospective Comparative Effectiveness Study of Electronic Health Records from 154 US Hospitals. Open Forum Infect. Dis..

[9] Maraolo A.E., Cascella M., Corcione S., Cuomo A., Nappa S., Borgia G., De Rosa F.G., Gentile I. (2017). Management of Multidrug-Resistant *Pseudomonas aeruginosa* in the Intensive Care Unit: State of the Art. Expert Rev. Anti Infect. Ther..

[10] Ng Q.X., Ong N.Y., Lee D.Y.X., Yau C.E., Lim Y.L., Kwa A.L.H., Tan B.H. (2023). Trends in *Pseudomonas aeruginosa (P. aeruginosa*) Bacteremia during the COVID-19 Pandemic: A Systematic Review. Antibiotics.

[11] Giacobbe D.R., Battaglini D., Enrile E.M., Dentone C., Vena A., Robba C., Ball L., Bartoletti M., Coloretti I., Di Bella S. (2021). Incidence and Prognosis of Ventilator-Associated Pneumonia in Critically Ill Patients with COVID-19: A Multicenter Study. J. Clin. Med..

[12] Schinas G., Polyzou E., Spernovasilis N., Gogos C., Dimopoulos G., Akinosoglou K. (2023). Preventing Multidrug-Resistant Bacterial Transmission in the Intensive Care Unit with a Comprehensive Approach: A Policymaking Manual. Antibiotics.

[13] Dalfino L., Stufano M., Bavaro D.F., Diella L., Belati A., Stolfa S., Romanelli F., Ronga L., Di Mussi R., Murgolo F. (2023). Effectiveness of First-Line Therapy with Old and Novel Antibiotics in Ventilator-Associated Pneumonia Caused by Carbapenem-Resistant *Acinetobacter baumannii*: A Real Life, Prospective, Observational, Single-Center Study. Antibiotics.

[14] Tiseo G., Brigante G., Giacobbe D.R., Maraolo A.E., Gona F., Falcone M., Giannella M., Grossi P., Pea F., Rossolini G.M. (2022). Diagnosis and management of infections caused by multidrug-resistant bacteria: Guideline endorsed by the Italian Society of Infection and Tropical Diseases (SIMIT), the Italian Society of Anti-Infective Therapy (SITA), the Italian Group for Antimicrobial Stewardship (GISA), the Italian Association of Clinical Microbiologists (AMCLI) and the Italian Society of Microbiology (SIM). Int. J. Antimicrob. Agents.

[15] Paul M., Carrara E., Retamar P., Tängdén T., Bitterman R., Bonomo R.A., de Waele J., Daikos G.L., Akova M., Harbarth S. (2022). European Society of Clinical Microbiology and Infectious Diseases (ESCMID) guidelines for the treatment of infections caused by multidrug-resistant Gram-negative bacilli (endorsed by European society of intensive care medicine). Clin. Microbiol. Infect..

[16] Gatti M., Pea F. (2023). Jumping into the future: Overcoming pharmacokinetic/pharmacodynamic hurdles to optimize the treatment of severe difficult to treat-Gram-negative infections with novel beta-lactams. Expert Rev. Anti Infect. Ther..

[17] Herrera F., Torres D., Laborde A., Berruezo L., Jordán R., Rossi I.R., Valledor A., Costantini P., Dictar M., Nenna A. (2023). Development of a Clinical Score to Stratify the Risk for Carbapenem-Resistant Enterobacterales Bacteremia in Patients with Cancer and Hematopoietic Stem Cell Transplantation. Antibiotics.

[18] Cano A., Gutiérrez-Gutiérrez B., Machuca I., Gracia-Ahufinger I., Pérez-Nadales E., Causse M., Castón J.J., Guzman-Puche J., Torre-Giménez J., Kindelán L. (2018). Risks of Infection and Mortality among Patients Colonized with *Klebsiella pneumoniae* Carbapenemase-Producing *K. pneumoniae*: Validation of Scores and Proposal for Management. Clin. Infect. Dis..

[19] Giannella M., Bartoletti M., Morelli M.C., Tedeschi S., Cristini F., Tumietto F., Pasqualini E., Danese I., Campoli C., Lauria N.D. (2015). Risk factors for infection with carbapenem-resistant *Klebsiella pneumoniae* after liver transplantation: The importance of pre- and posttransplant colonization. Am. J. Transplant..

[20] Kitaya S., Kanamori H., Katori Y., Tokuda K. (2023). Impact of Persistent Multidrug-Resistant Gram-Negative Bacteremia on Clinical Outcome and Mortality. Antibiotics.

[21] Kitaya S., Kanamori H., Baba H., Oshima K., Takei K., Seike I., Katsumi M., Katori Y., Tokuda K. (2023). Clinical and Epidemiological Characteristics of Persistent Bacteremia: A Decadal Observational Study. Pathogens.

[22] López-Cortés L.E., Del Toro M.D., Gálvez-Acebal J., Bereciartua-Bastarrica E., Fariñas M.C., Sanz-Franco M., Natera C., Corzo J.E., Lomas J.M., Pasquau J. (2013). Impact of an evidence-based bundle intervention in the quality-of-care management and outcome of *Staphylococcus aureus* bacteremia. Clin. Infect. Dis..

[23] Huttner B.D., Sharland M., Huttner A. (2023). On culture and blood cultures. Clin. Microbiol. Infect..

[24] Kitaya S., Kanamori H., Katori Y., Tokuda K. (2023). Clinical Characteristics and Outcomes of Persistent Staphylococcal Bacteremia in a Tertiary Care Hospital. Antibiotics.

[25] Kuehl R., Morata L., Boeing C., Subirana I., Seifert H., Rieg S., Kern W.V., Kim H.B., Kim E.S., Liao C.H. (2020). Defining persistent *Staphylococcus aureus* bacteraemia: Secondary analysis of a prospective cohort study. Lancet Infect. Dis..

[26] Lemonnier D., Machuel M., Obin O., Outurquin G., Adjidé C., Mullié C. (2023). Trends in Antibiotic-Resistant Bacteria Isolated from Screening Clinical Samples in a Tertiary Care Hospital over the 2018–2022 Period. Antibiotics.

